# Association Between Polyp Size and Malignant Invasion in Colorectal Polyps <20 mm: A Single-Trust Observational Study

**DOI:** 10.7759/cureus.115679

**Published:** 2026-09-02

**Authors:** Mahmud Riad, Mohammad Hossain, Mohamed Hassan, Sneha Gupta, Elisabeth Bonor, Maliha Ata, Amr K Ebrahim, Hatim Albirnawi, Sanjay Joshi, Raza Moosvi

**Affiliations:** 1 Colorectal Surgery, Tunbridge Wells Hospital, Tunbridge Wells, GBR; 2 General Surgery, Maidstone and Tunbridge Wells NHS Trust, Tunbridge Wells, GBR; 3 Surgery, Maidstone and Tunbridge Wells NHS Trust, Tunbridge Wells, GBR; 4 Pharmacology and Therapeutics, Chattagram Maa-O-Shishu Hospital Medical College, Chattogram, BGD; 5 Surgery, Maidstone and Tunbridge Wells NHS Trust, Maidstone, GBR; 6 General Surgery, Cairo University, Cairo, EGY; 7 Upper GI Surgery, Maidstone and Tunbridge Wells NHS Trust, Maidstone, GBR; 8 General Surgery, Maidstone and Tunbridge Wells NHS Trust, Kent, GBR

**Keywords:** colonoscopy, colorectal polyp, endoscopy, malignancy threshold, polypectomy, tattooing

## Abstract

Introduction

Colorectal polyps are precursors to colorectal cancer. Accurate identification and follow-up of polyps during colonoscopy depend on reliable localization techniques, including tattooing. However, the optimal minimum polyp size threshold for routine tattooing remains poorly defined in current clinical guidelines. This study evaluated the association between polyp size and malignant invasion among colorectal polyps measuring smaller than 20 mm, with consideration of its implications for lesion localization and tattooing.

Methods

A retrospective observational study was conducted at Maidstone and Tunbridge Wells (MTW) NHS Trust. Data from 450 patients who underwent polypectomy of colorectal polyps < 20 mm during colonoscopy or flexible sigmoidoscopy between September 2021 and September 2022 were collected. One index lesion per patient was analyzed. In patients with multiple eligible polyps, the index lesion was selected according to a predefined hierarchy based on malignant invasion, dysplasia grade, size, and chronological order. Patients with polyps ≥ 20 mm and known hereditary colorectal polyposis syndromes were excluded. Chi-square tests were performed using SPSS version 25.0 (IBM Corp., Armonk, NY).

Results

A total of 450 participants were enrolled, including 288 (64.0%) male participants and 162 (36.0%) female participants. Polyp size distribution was 190 (42.2%) for 0-9 mm and 260 (57.8%) for 10-19 mm. Only 36 (8.0%) polyps were tattooed. Polyp locations included the right hemicolon in 134 patients (29.78%), transverse colon in 92 (20.4%), left hemicolon in 41 (9.1%), sigmoid colon in 128 (28.4%), and rectum in 55 (12.22%). Pedunculated polyps were identified in 143 patients (31.8%), whereas 256 (56.9%) had sessile polyps. Histologically, 251 polyps (55.8%) were tubular adenomas, and 349 (77.6%) exhibited low-grade dysplasia. Malignant invasion was detected in 27 patients (6%).

Conclusion

Malignant invasion was significantly more frequent in larger polyps within the <20 mm cohort. However, the low event rate among smaller lesions makes it difficult to define a reliable minimum size for tattooing. Larger prospective multicenter studies are therefore needed.

## Introduction

Colorectal cancer (CRC) is one of the most prevalent malignancies globally, and adenomatous polyps are major precursor lesions in its development [1,2]. Early detection and complete excision of colorectal polyps during colonoscopy are the cornerstone of prevention of this malignancy [3]. When histological examination reveals malignant invasion or high-risk findings requiring additional intervention, precise identification of polypectomy sites is crucial for subsequent surveillance and surgical planning [4]. Endoscopic tattooing, the submucosal injection of an indelible dye such as India ink or carbon suspension, is widely used to mark polypectomy sites, enabling endoscopists and surgeons to relocate lesions on repeat examination or at surgery [5]. This technique plays a pivotal role in modern gastrointestinal practice by improving lesion localization and intraoperative guidance. Despite its routine use, current guidelines recommend tattooing suspicious-appearing lesions during colonoscopy, regardless of polyp size. According to the British Society of Gastroenterology (BSG) guidelines, polyps measuring 20 mm or more should be tattooed, except those located in the rectum or cecum [6]. A polyp size threshold of ≥10 mm has been proposed as an indication for tattooing [7]. However, tattooing practices remain variable and may be influenced by institutional protocols and endoscopist preference [8]. Such inconsistency may arise from variations in institutional protocols, endoscopist preference, and the absence of robust evidence establishing a universally accepted lower size cutoff for tattooing.

Determining the optimal lower threshold for tattooing is clinically important for endoscopy. Application of tattooing below a clinically relevant threshold can lead to unnecessary endoscopic procedures. In contrast, omission of tattooing in a lesion, subsequently confirmed as malignant, may make intraoperative localization more difficult, delay appropriate oncological management, and adversely influence patient outcomes while also increasing demands on healthcare resources [7,9].

Several studies have attempted to define size-based criteria, but evidence from a single-trust, population-representative cohort remains limited [7,8]. This retrospective single-center study aimed to evaluate the association between polyp size and malignant invasion among colorectal polyps measuring <20 mm.

## Materials and methods

Study design and setting

This was a retrospective, single-center observational study conducted at Maidstone and Tunbridge Wells (MTW) NHS Trust, United Kingdom. Ethical approval was obtained from the ethical review board of MTW NHS Trust (reg no.: 1512-23/24).

Patient selection

Patients undergoing polypectomy for a polyp <20 mm at colonoscopy or flexible sigmoidoscopy within MTW NHS Trust between September 2021 and September 2022 were identified from the endoscopy reporting system.

Inclusion Criteria

A “single patient, single polyp” inclusion criterion was applied. Patients with multiple polyps were included, but only one index lesion per patient was analyzed. In patients with more than one eligible lesion, a single index polyp was selected according to a predefined hierarchy. Lesions with malignant invasion were prioritized; in the absence of malignancy, the lesion with the highest grade of dysplasia was selected; when the dysplasia grade was equivalent, the largest lesion was chosen; and if a tie remained, the first chronologically recorded lesion was included.

Exclusion Criteria

Polyps measuring ≥20 mm and patients with known hereditary colorectal polyposis syndromes, including familial adenomatous polyposis (FAP) and Lynch syndrome, were excluded. For patients with multiple eligible polyps, lesions other than the predefined index polyp were excluded from the analysis.

Data collection

Demographic data (age and sex), procedural data (polyp size, location, morphology, and tattooing status), and histopathological data (histological subtype, dysplasia grade, and malignant invasion) were collected from electronic patient records. Polyp size was recorded from the endoscopy report and represented the endoscopist’s estimated size at the time of the procedure. Malignant invasion was defined as histologically confirmed invasive adenocarcinoma within the polyp. Non-neoplastic lesions were retained because lesion eligibility was determined at endoscopy before histopathological diagnosis was available, thereby reflecting routine clinical practice and avoiding post-histology selection of the cohort.

Statistical analysis

The primary endpoint of the study was malignant invasion on histopathological assessment. Secondary outcomes included histological subtype, dysplasia grade, tattooing status, and associations between polyp size group and clinicopathological characteristics. Data were analyzed using SPSS version 25.0 (IBM Corp., Armonk, NY). Categorical variables were expressed as frequencies and percentages, and associations were assessed using the chi-square test. A p-value < 0.05 was considered statistically significant.

## Results

Study population

A total of 450 patients were included in this study. Among them, 288 (64%) were male participants, and 162 (36%) were female participants.

Polyp characteristics

The polyps ranged in size from 0 to 19 mm. Most were 10-19 mm in diameter (n = 260, 57.8%), while 190 (42.2%) were 0-9 mm. Only 36 (8%) polyps in the cohort had been tattooed at the time of the index procedure.

Polyps were distributed throughout the colorectum, most commonly in the right hemicolon (29.7%) and sigmoid colon (28.4%), followed by transverse colon (20.4%), rectum (12.2%), and left hemicolon (9%). Sessile morphology was the most frequent (n = 256, 56.9%), followed by pedunculated morphology (n = 143, 31.8%), while the remainder were flat or of other morphological types (Table 1).

**Table 1:** Baseline characteristics of the polyp Percentages may not total 100% due to rounding.

Variable	Category	Frequency (n = 450)	Percentage (%)
Poly size (mm)	0-9 mm	190	42.2
	10-19 mm	260	57.8
Tattooing at index procedure	Yes	36	8
	No	414	92
Polyp location	Right hemicolon	134	29.8
	Transverse colon	92	20.5
	Left hemicolon	41	9.1
	Sigmoid colon	128	28.4
	Rectum	55	12.2
Polyp morphology	Pedunculated	143	31.8
	Sessile	256	56.9
	Flat	2	0.4
	Sessile serrated	2	0.4
	Sub-pedunculated	47	10.4

Histology outcomes 

Tubular adenomas were identified in 251 (55.8%) patients and were the most prevalent form of polyp. In general, tubular adenomas are considered to have a low chance of developing into cancer. Tubulo-villous adenoma was the second most frequent histology, identified in 124 patients (27.6%). Hyperplastic polyps were identified in 47 (10.4%) patients. Sessile serrated lesions (SSL) were found in 14 patients (3.1%). The remaining histology, which included villous adenomas, serrated adenomas, inflammatory polyps, and normal polyps, was all detected in extremely tiny numbers, affecting just one to four (0.2%-0.9%) patients (Table 2).

**Table 2:** Distribution of histological types by polyp size group

Histological type	0–9 mm, n (%)	10–19 mm, n (%)	Total, n
Hyperplastic polyp	36 (18.9)	11 (4.2)	47
Inflammatory polyp	1 (0.5)	3 (1.2)	4
Normal polyp	1 (0.5)	0 (0.0)	1
Serrated adenoma	1 (0.5)	3 (1.2)	4
Serrated lesion	0 (0.0)	1 (0.4)	1
Serrated polyp	0 (0.0)	1 (0.4)	1
Sessile serrated lesion	6 (3.2)	8 (3.1)	14
Tubular adenoma	131 (68.9)	120 (46.2)	251
Tubulo-villous adenoma	14 (7.4)	110 (42.3)	124
Villous adenoma	0 (0.0)	3 (1.2)	3
Total	190 (100)	260 (100)	450

Dysplasia grade

Low-grade dysplasia was present in 349 (77.6%) cases, whereas high-grade dysplasia and no dysplasia were present in 30 (6.7%) and 71 (15.8%) cases, respectively (Table 3).

**Table 3:** Dysplasia grade of polyp Percentages may not total 100% due to rounding.

Dysplasia grade	Frequency (n = 450)	Percentage (%)
Low-grade dysplasia	349	77.6
High-grade dysplasia	30	6.7
No dysplasia	71	15.8

Malignant invasion

Malignant invasion was observed in 27 patients (6%), whereas 423 (94%) did not exhibit any features of malignant infiltration. Malignant invasion was observed in 25 of 260 polyps in the 10-19 mm group compared with 2 of 190 polyps in the 0-9 mm group, corresponding to an unadjusted odds ratio of approximately 10.0. These findings suggest that larger polyps within the <20 mm cohort were associated with markedly increased odds of malignant invasion (Table 4).

**Table 4:** Association between polyp size group and clinicopathological characteristics Values are derived from Pearson’s χ² test. Polyp size was categorized as 0-9 mm and 10-19 mm. Sparse cell counts were present for some variables; therefore, these associations should be interpreted with caution.

Clinicopathological characteristics	χ² value	df	p-value
Polyp location	67.665	4	<0.001
Polyp morphology	59.367	4	<0.001
Tattooing status	21.565	1	<0.001
Histological type	87.620	9	<0.001
Dysplasia grade	33.642	2	<0.001
Malignant invasion	14.281	9	<0.001

Association between polyp size group and clinico-pathological characteristics

Categorical associations with polyp size group were assessed using Pearson’s χ² test. Polyp size category was significantly associated with polyp location, morphology, tattooing status, histological subtype, dysplasia grade, and malignant invasion (Table 4). The location of the polyp within the colon is significantly associated with the size of the polyps smaller than 20 mm.

## Discussion

This retrospective observational study from a single trust evaluated the association between polyp size and malignant invasion among colorectal polyps measuring <20 mm, with consideration of the implications for lesion localization and tattooing.

The current study included 450 patients who underwent polypectomy for polyps measuring <20 mm. The cohort had a male predominance, with 288 (64.0%) male and 162 (36.0%) female patients, similar to the sex distribution observed in a study from Turkey [10]. Evidence from these two studies suggests that men may have a higher prevalence of colorectal polyps and tumors than women [11].

An Iranian population-based study reported a higher prevalence of right-sided colonic polyps, consistent with the findings of the present study, which also demonstrated a predominance of polyps in the right hemicolon [12]. In the present study, 128 (28.4%) polyps were located in the sigmoid colon. A similar finding was reported in another study, in which 24.70% of colorectal polyps were located in the sigmoid colon [13]. The present study demonstrated a significant association between polyp size category and morphology. Since both size and morphology are clinically relevant features in predicting histological behavior and malignant potential, their combined evaluation may help guide appropriate management decisions. This finding is consistent with previous evidence, including the study by Atalaia-Martins et al., which also reported an association between polyp morphology and size [14]. Malignant invasion and lymph node involvement are usually seen in larger lesions [15-17]. The findings of the present study are consistent with this observation, as polyp size was found to be significantly associated with malignant invasion. Two findings are important in this study. First, most malignant lesions in this <20 mm cohort were 10 mm or larger. Second, not all malignant lesions were 10 mm or larger; two lesions with malignant invasion measured 8 mm and 9 mm.

The clinical rationale for tattooing is practical rather than biological. Tattooing does not alter malignant potential, but it facilitates lesion localization when histology reveals invasive cancer or other high-risk features [3,18]. This becomes especially important if the original resection site is no longer easily visible, if laparoscopic surgery is planned, or if repeat endoscopy is needed to assess residual or recurrent tissue [3,5,7]. Within this cohort, tattooing was performed in only 8.0% of lesions measuring <20 mm, highlighting possible inconsistency in routine localization practice.

The presence of malignant invasion in 27 (6.0%) polyps measuring <20 mm is clinically important. A noteworthy finding of this study was the presence of malignant invasion in two polyps measuring ≤9 mm. Although small polyps generally have a lower risk of malignant invasion, they should not be considered entirely benign based solely on their size. Avoidance of tattooing in such lesions may increase challenges in subsequent clinical management.

The findings also align with the broader principle that polyp size correlates with the risk of advanced neoplasia [19]. However, size alone should not dictate management. Endoscopic appearance, morphology, location, and technical confidence in future site recognition remain important [3,5,7,8]. The anticipated need for future localization should also be considered in clinical management rather than relying on size alone.

Current BSG guidance recommends tattooing lesions ≥20 mm and lesions suspicious for malignancy outside the rectum and caecum, while smaller lesions should be assessed according to their endoscopic risk features [20]. In the present cohort, malignant invasion was more frequent in the 10-19 mm group; however, two malignant lesions were also identified in the 0-9 mm group. This finding highlights that small size alone does not exclude malignancy and that smaller lesions with suspicious endoscopic features or a potential need for future localization may still warrant consideration for tattooing. However, the present data do not support a new size-based tattooing threshold.

This study has several limitations. First, its retrospective, single-center design may limit the generalizability of the findings. Second, polyp size was based on the endoscopist's estimated measurement at the time of the procedure, which may introduce measurement variability. In addition, although patients with multiple polyps were eligible, only one predefined index lesion per patient was included in the analysis. This may not fully reflect routine colonoscopy practice, where multiple polyps are often found, and management decisions may depend on the features of more than one lesion. The predefined selection process may also have resulted in more advanced lesions being included as the index lesion. Therefore, future prospective multicenter studies are needed to further evaluate the association between polyp size and malignant invasion.

## Conclusions

In this cohort of colorectal polyps measuring <20 mm, larger polyp size was significantly associated with malignant invasion. However, the small number of malignant events among smaller polyps prevents determination of a reliable lower size threshold for tattooing. Further studies are needed to better characterize the relationship between polyp size and malignant invasion.
